# Expression and clinical significance of growth differentiation factor-15 in vascular cognitive impairment based on bioinformatic analysis

**DOI:** 10.1515/jtim-2026-0004

**Published:** 2026-02-13

**Authors:** Jiaqi Lin, Ning Mao, Zheng Wu, Rui Yang, Yanli Zhang, Yusong Ge

**Affiliations:** Department of Neurology, The Second Hospital of Dalian Medical University, Dalian, Liaoning Province, China; Department of Neurology, Zhongshan Hospital of Dalian University, Dalian, Liaoning Province, China

## To the editor

As China’s population ages, acute ischemic stroke (AIS) and dementia are becoming increasingly prevalent and significantly impacting the quality of life of the elderly.^[1]^ Aberrant upregulation of growth differentiation factor-15 (GDF-15) has been associated with the development of AIS and Alzheimer’s disease (AD).^[2,3]^ However, its role in vascular cognitive impairment (VCI) remains unclear. This study employs bioinformatics for an initial exploration, followed by serum-level validation, to systematically investigate the expression features of GDF-15 in VCI patients, and to evaluate its potential as a prognostic and diagnostic biomarker.

**Figure 1:**
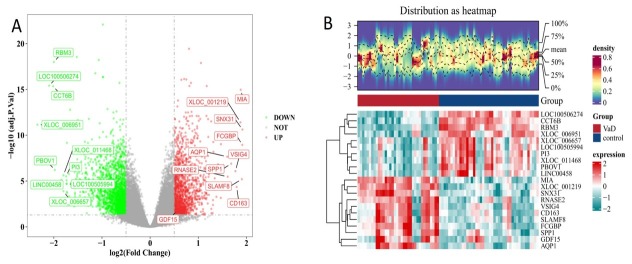
DEGs between VCI patients and NC. (A) Volcano plots. (B) Heatmap. Red indicates a relative upregulation of gene expression, and green indicates a relative downregulation of gene expression. DEGs: differentially expressed genes; VCI: vascular cognitive impairment; NC: normal control.

The datasets required for our study were obtained from the Gene Expression Omnibus (GEO) database. The VCI dataset was retrieved by citing the following PMID: 30990880 (GSE122063). The ischemic stroke dataset was retrieved by citing PMID: 25036109 (GSE58294). Differentially expressed genes (DEGs) between healthy individuals and patients with VCI in the GSE122063 dataset were analyzed using the limma package. GDF15 was one of the DEGs identified, showing upregulation (|log_₂_FC| > 0.5 and adj.P.Val < 0.05), as shown in Figure 1. Gene expression differences between healthy individuals and AIS patients were analyzed based the GSE58294 dataset. No significant difference in GDF15 levels between AIS patients and healthy individuals was found in our results, as shown in Supplementary Figure S1.

A GDF-15 transcription factor regulatory network map was constructed using the NetworkAnalyst tool to predict transcription factors (TFs) associated with GDF-15, as shown in Supplementary Figure S2. Further GO/KEGG pathway enrichment analysis was conducted to elucidate the potential pathological mechanisms by which GDF-15 contributes to VCI. The results revealed that GDF-15 is involved in the formation of histone deacetylase complex, SWI/SNF superfamily-type complex and polycomb repressive complexes. GDF-15 is also implicated in various biological processes, including histone modification, intracellular receptor signaling, ATP-dependent chromatin remodeling, and the transforming growth factor-β (TGF-β) signaling pathway. These results are presented in Supplementary Figure S3.

Based on the results of the bioinformatics analysis, the levels of GDF-15 in human serum were assessed to confirm its differential expression in VCI. A total of 90 patients who attended the Second Hospital of Dalian Medical University between August 30, 2018 and December 31, 2019 were enrolled in this study. Basic information, hematological test results, and 3 mL of fasting blood collected in the early morning were obtained from all participants. The study was approved by the Ethics Committee of the Second Affiliated Hospital of Dalian Medical University (Ethics Review Approval Number: KY2025-237-01), and all participants provided informed consent and completed the necessary documentation. Detailed inclusion criteria are provided in Supplementary Tables S1 and S2. All study participants had consistent and homogeneous demographic information and blood collection results, as presented in Supplementary Table S3. All participants were evaluated by professionally trained neuropsychological assessors. Specific scale items and results are presented in Supplementary Table S4.

PASS software 2020 (NCSS LLC., Kaysville, Utah, USA) was used to estimate the sample size (α = 0.05, β = 0.1). Employing a balanced randomization scheme, the final sample comprised 30 AIS patients, 30 VCI patients, and 30 normal controls (NC), adjusted for test performance and potential dropouts. Count data were analyzed using chi-square tests (*P* < 0.05) with Bonferroni correction. Continuous data were analyzed using one-way analysis of variance (ANOVA; *P* < 0.05) and nonparametric tests, with Bonferroni correction (*P* < 0.05). The diagnostic utility of GDF-15 was assessed using receiver operating characteristic (ROC) curves and the area under the curve (AUC). Pearson’s correlation analysis was applied to continuous variables with normal distribution, and a correlation heatmap was generated. Significant differences in serum GDF-15 levels were observed between the VCI, AIS, and NC groups (*P* < 0.05). The VCI group exhibited the highest serum GDF-15 concentration, followed by the AIS group. The NC group exhibited the lowest concentration, as shown in Supplementary Figure S4. Correlation analysis revealed negative correlations between GDF-15 levels and mini-mental state examination (MMSE, R = -0.53, *P* < 0.05), montreal cognitive assessment (MoCA; R = -0.55, *P* < 0.05) and activity of daily living (ADL; R = -0.56, *P* < 0.05) scores, as shown in Supplementary Figure S5. ROC curve analysis revealed that the AUC for diagnosing VCI using GDF-15 was 0.923 (95% confidence interval: 0.860-0.987, *P* < 0.01), exceeding 0.800 and indicating high diagnostic accuracy for serum GDF-15 levels with a threshold of 9.10 ng/mL. Additionally, the AUC for diagnosing AIS using GDF-15 was 0.765 (95% confidence interval: 0.647-0.883, *P* < 0.01), with a threshold of 5.73 ng/mL, as shown in Supplementary Figure S6.

Based on the discovery of GDF-15-related cellular components and biological pathways, and a review of the literature, we propose that GDF-15 may be involved in the development and progression of VCI *via* the following potential mechanisms. Post-translational modifications (PTMs) of histones, such as acetylation and methylation, play a significant role in synaptic plasticity and memory formation.^[4]^ ATP-dependent chromatin remodeling mechanisms dynamically regulate chromatin structure by altering the position and composition of nucleosomes. Studies have demonstrated that this process is pivotal in the formation of long-term memory during development and in the brain, as it regulates gene expression, plasticity, and cellular function.^[5]^ TGF-β plays a central role in various aspects of neural system development and function. These include the initial formation of the embryonic nervous system, the growth, proliferation, migration, and differentiation of neurons and glial cells, modulation of inflammatory responses in the nervous system, regulation of excitatory and inhibitory synaptic transmission, and influence on synaptic plasticity and memory in animals.^[6]^ The Polycomb repressive complex 2 (PRC2) contains four main core subunits: EZH1/2, SUZ12, EED, and RBP. EZH1/2 plays a pivotal role in regulating chromatin modification by dimethylating and trimethylating histone H3 at lysine 27 (H3K27me2/3). Previous studies have shown that histone modifications play a significant role in the formation of long-term memory (LTM). Epigenetic mechanistic studies have demonstrated that EZH2 mediates the ubiquitination and subsequent proteasomal degradation of aggregated α-synuclein-phosphatidylserine 129 (pSer129), which exhibits neuroprotective properties, such as anti-inflammation, reduction of oxidative stress, and prevention of apoptosis.^[7]^

In summary, GDF-15 plays a significant role in regulating memory development, neuronal differentiation, and synapse formation. Furthermore, it may participate in the modulation of glial cell activation and neuroinflammation while preserving the integrity and function of the blood-spinal cord barrier (BSCB) and the blood-brain barrier (BBB).^[8]^ The pathogenesis of VCI is well established to be closely associated with the neurovascular unit, disruption of the BBB, mitochondrial energy metabolism abnormalities, excessive microglial activation, neuroinflammation, and oxidative stress-induced damage.^[9,10]^ Our study found that GDF-15 expression levels were elevated in VCI patients, suggesting that this factor may exert neuroprotective effects through mechanisms such as alleviating oxidative stress, regulating memory processes, stabilizing the BBB, and promoting neuronal differentiation. Additionally, the genome-wide results from GSE58294 did not align with the proteomics findings. We speculate that this discrepancy may be due to differences in the patient populations: the GSE58294 dataset primarily included patients with cardioembolic stroke, which is often associated with large vessel injury. In contrast, our study predominantly involved strokes originating from atherosclerotic plaque rupture or thrombosis. These variations in baseline characteristics between the two cohorts likely contributed to the mismatch. Furthermore, all patients in the GSE58294 dataset received treatment within three hours, whereas some patients in this study did not. Post-treatment repair of ischemic injury may have influenced expression levels.

This study provides initial insights into the role of GDF-15 in VCI, but it has limitations. The bioinformatic analysis does not employ advanced techniques, such as protein-protein interaction (PPI) network analysis, and the proposed protective role of GDF-15 remains speculative without experimental confirmation. Additionally, the clinical relevance is constrained by the absence of comparisons with other cognitive disorders. Future research should incorporate more sophisticated analytical approaches and experimental validation to establish GDF-15 as a biomarker for VCI and enable broader comparative studies.

## Supplementary Material

Supplementary Material Details
