# Supplementary material for: Expression and clinical significance of growth differentiation factor-15 in vascular cognitive impairment based on bioinformatic analysis
Source: J Transl Int Med. 2026 Feb 13;14(1):150–3. doi: 10.1515/jtim-2026-0004 (PMC12916273; doi:10.1515/jtim-2026-0004)
Supplement: Supplementary file 1 — Supplementary Material Details [file jtim-2026-0004_sm.pdf]

Supplementary materials

Supplementary Table S1: Study participant enrollment criteria

Group	Enrollment criteria
VCI group (n = 30)	(1) Age ⩾ 18 years.<br>(2) All individuals fulfilled the diagnostic criteria for VCI as outlined in the 2014 International Society for VASCOG and the 2015 Chinese guidelines for diagnosing and treating dementia and cognitive disorders.<br>(3) The neuropsychological assessment verified the existence of cognitive dysfunction, indicated by a MMSE score ⩽ 26 and a CDR score ⩾ 0.5.<br>(4) Clinical symptoms or imaging manifestations were consistent with the diagnosis of cerebrovascular disease, and there was a connection between cerebrovascular illness cognitive dysfunction.
AIS group (n = 30)	(1) Age ⩾ 18 years.<br>(2) The patients' symptoms, indicators (such as weakness on one side of the body, paralysis of the facial muscles, loss of sensation, visual field loss, difficulty speaking, and others), and neurological examination were indicative of AIS. Brain MRI confirmed the presence of acute cerebral infarction.<br>(3) The MMSE score > 26, and the CDR score is = 0.
Control group (n = 30)	(1) Age ⩾ 18 years.<br>(2) No previous history of cerebrovascular illness.<br>(3) MRI structural imaging did not detect any visible abnormalities, such as multiple lacunar cerebral infarctions, critical cerebral infarction, or white matter lesions.<br>(4) The MMSE score > 26, and the CDR score = 0.

VCI: vascular cognitive impairment; AIS: acute ischemic stroke; MRI: magnetic resonance imaging; VASCOG: Vascular Behavioral and Cognitive Disorders; MMSE: Mini-Mental State Examination; CDR: Clinical Dementia Rating.

Supplementary Table S2: Study participant exclusion criteria

**Group**	**Exclusion criteria**
VCI group (*n* = 30)	(1) Participants unable to undergo the test and clinical evaluation due to language dysfunction, visual or hearing impairment, or other symptoms.<br>(2) Participants had a prior medical background of schizophrenia, intense anxiety, depression, bipolar disorder, and other psychiatric conditions.<br>(3) Participants with cognitive dysfunction caused by other factors, such as Alzheimer's disease, Parkinson's disease, frontotemporal dementia, normal pressure hydrocephalus, central nervous system trauma, hypothyroidism, tumors, infections and metabolic disorders.<br>(4) Previous medical conditions included severe cardiac, hepatic and renal insufficiency, as well as hematological disorders.
AIS group (*n* = 30)	(1) Patients had a history of cerebrovascular illness for more than two weeks.<br>(2) All other exclusion criteria were the same as those for the VCI group.
Control group (*n* = 30)	(1) Participants had no history of cerebrovascular disease or cognitive impairment.<br>(2) All other exclusion criteria were the same as those for the VCI group.

VCI: vascular cognitive impairment; AIS: acute ischemic stroke; MRI: magnetic resonance imaging.

Supplementary Table S3: Data from VCI group, ACI group, and Control group

**Item**	**Control group (*n* = 30)**	**AIS group (*n* = 30)**	**VCI group (*n* = 30)**	χ^2/F	***P* value**
Gender (male/female)	13/17	15/15	15/15	0.356	0.837
Age (year)	64.9 ± 9.1	67.2 ± 10.0	70.0 ± 10.0	2.078	0.131
BMI (kg/m^2)	23.7 ± 2.8	23.8 ± 3.0	23.5 ± 3.0	0.093	0.930
Hypertension (yes/no)	15/15	21/9	22/8	4.170	0.124
Diabetes (yes/no)	9/21	15/15	8/22	4.170	0.124
Smoking (yes/no)	7/23	9/21	10/20	0.757	0.685
TC (mmol/L)	4.36 ± 1.11	4.59 ± 1.15	4.77 ± 1.29	0.900	0.410
TG (mmol/L)	1.67 ± 1.10	1.57 ± 1.77	1.68 ± 1.12	0.065	0.937
FBG (mmol/L)	6.17 ± 2.20	6.72 ± 2.34	6.33 ± 1.92	0.513	0.600
Hb1Alc (%)	6.14 ± 1.47	6.72 ± 1.54	6.33 ± 1.16	1.294	0.280
Cr (μmol/L)	68.28 ± 19.17	80.18 ± 53.10	69.89 ± 22.78	1.012	0.368
UA (μmol/L)	362.81 ± 81.85	318.62 ± 84.73	323.51 ± 99.00	2.230	0.114
AST (U/L)	21.65 ± 6.38	23.10 ± 6.13	22.85 ± 10.33	0.289	0.750
ALT (U/L)	21.04 ± 9.42	24.66 ± 10.77	27.47 ± 18.54	1.703	0.188

VCI: vascular cognitive impairment; AIS: acute ischemic stroke; BMI: Body Mass Index; TC:

Total Cholesterol; TG: Triglycerides; FBG: Fasting Blood Glucose; Cr: Creatinine; UA: Uric Acid; AST: Aspartate Aminotransferase; ALT: Alanine Aminotransferase.

Supplementary Table S4: Neuropsychological tests of the study subjects

**Test**	**Control group (*n* = 30)**	**AIS group (*n* = 30)**	**VCI group (*n* = 30)**	**χ^2/F**	***P* value**
MMSE	28.90 ± 0.96	28.47 ± 1.04	18.87 ± 6.03*#	75.39	< 0.01
MoCA	28.67 ± 1.06	28.37 ± 1.07	14.43 ± 5.65*#	127.98	< 0.01
ADL	98.83 ± 3.13	92.33 ± 5.98	53.174 ± 20.06*#	119.15	< 0.01
CDR	0.00 ± 0.00	0.00 ± 0.00	1.26 ± 0.76*#	82.06	< 0.01

$^*P < 0.01$ *vs*. Control group; $^\#P < 0.01$ *vs*. AIS group. VCI: vascular cognitive impairment; AIS: acute ischemic stroke; MMSE: Mini-Mental State Examination; MoCA: montreal cognitive assessment; ADL: activity of daily living; CDR: clinical dementia rating.

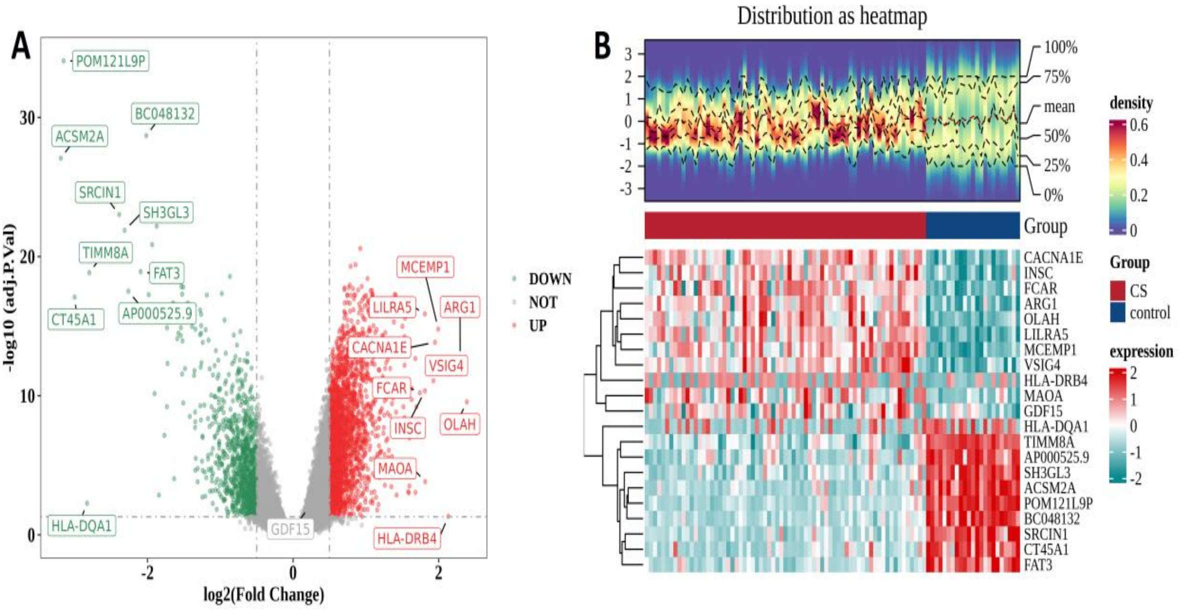

Supplementary Figure S1: DEGs between AIS patients and NC. (A) Volcano plots. (B) Heatmap. Red indicates a relative upregulation of gene expression, green indicates a

relative downregulation of gene expression, and gray indicates no differential gene expression. DEG: differentially expressed genes; AIS: acute ischemic stroke; NC: normal control.

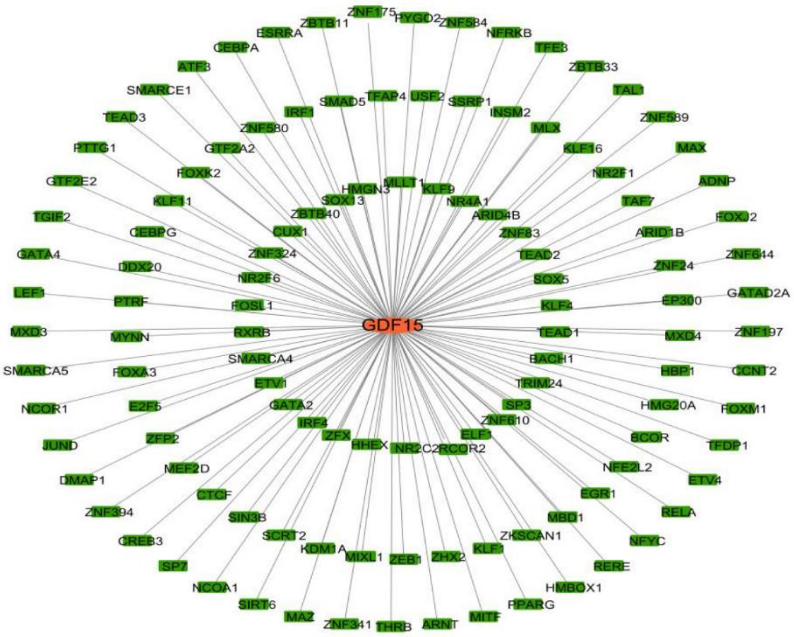

Supplementary Figure S2: GDF15-TFs network graph includes a total of 121 points and 120 edges.

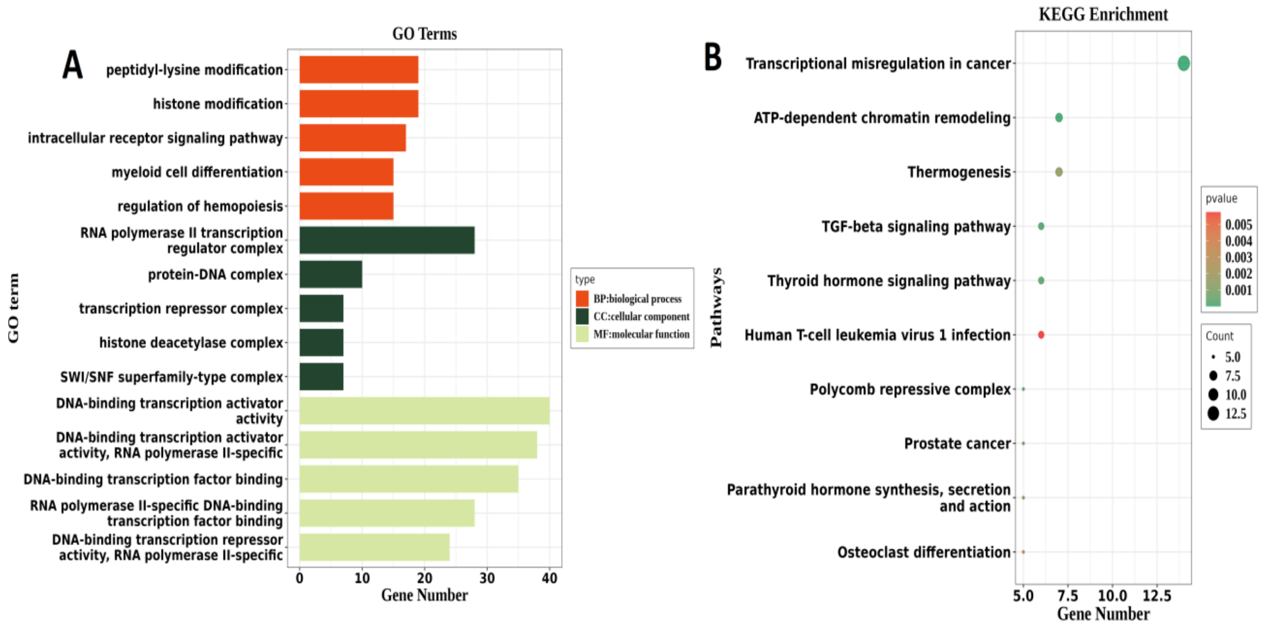

Supplementary Figure S3: Enrichment pathway analysis of DEGs. (A) GO enrichment analysis. Top 10 enriched pathways for BP, CC and MF. The length of the bar indicates the number of genes expressed in the pathway and the color indicates the *P* value. (B) KEGG enrichment analysis. Top 10 KEGG-enriched pathways, the size of the dots indicates the number of genes expressed in the pathway, and the color shade indicates the *P* value. DEG: differentially expressed genes; GO: Gene Ontology; BP: biological process; CC: cell component; MF: molecular function; KEGG: Kyoto Encyclopedia of Genes and Genomes.

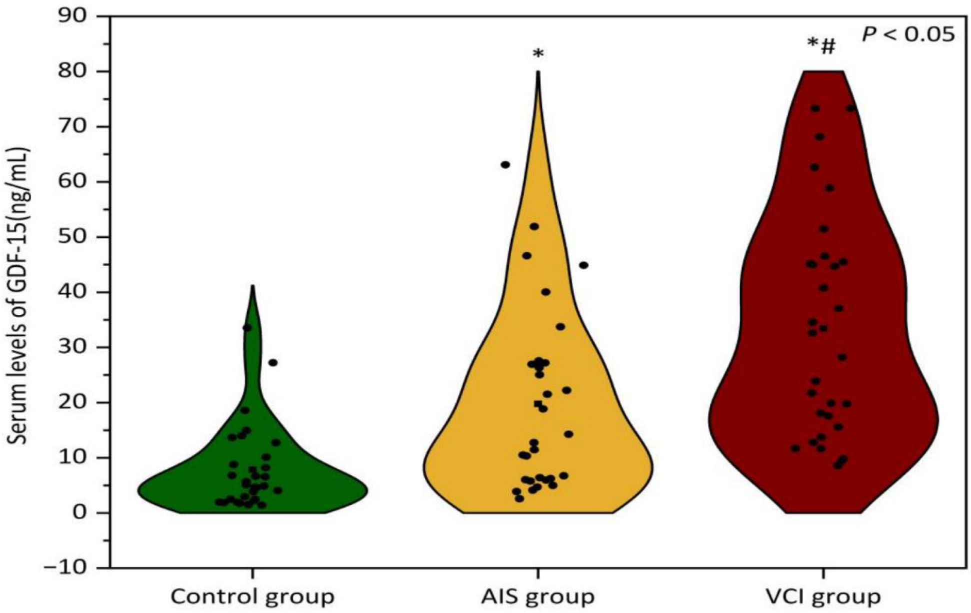

Supplementary Figure S4: Concentration of GDF-15 in three groups. ⋆ Statistically significant compared to the Control group (*P* < 0.05); # Statistically significant compared to the AIS group (*P* < 0.05). GDF-15: growth differentiation factor 15; AIS: acute ischemic stroke; VCI: vascular cognitive impairment.

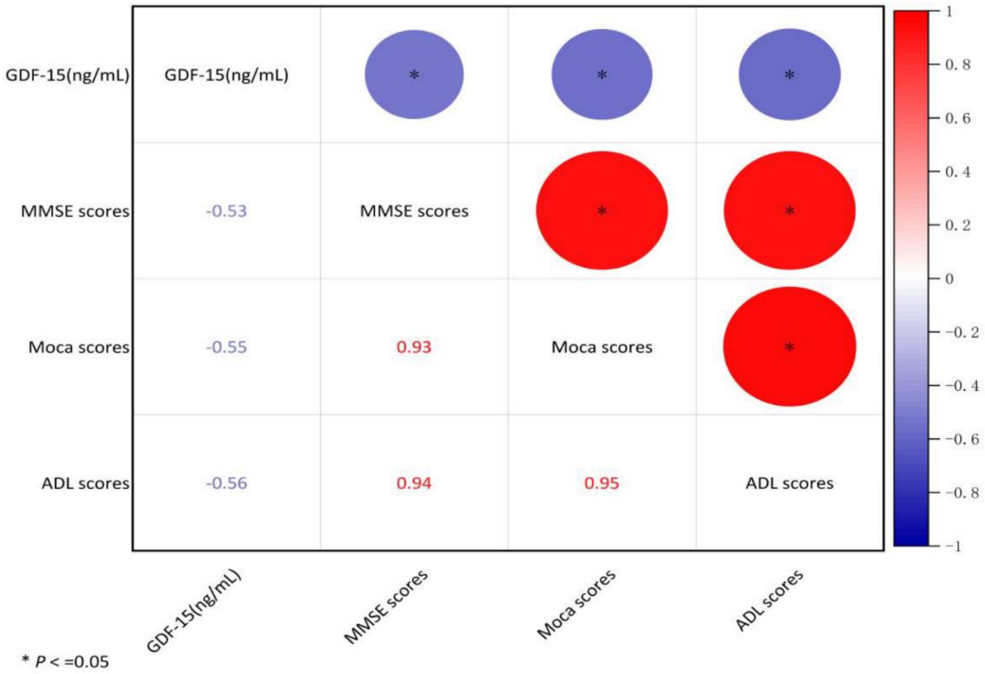

Supplementary Figure S5: A correlation heat map was plotted of GDF-15. GDF-15: growth differentiation factor 15; MMSE: Mini-Mental State Examination; MoCA: montreal cognitive assessment; ADL: activity of daily living.

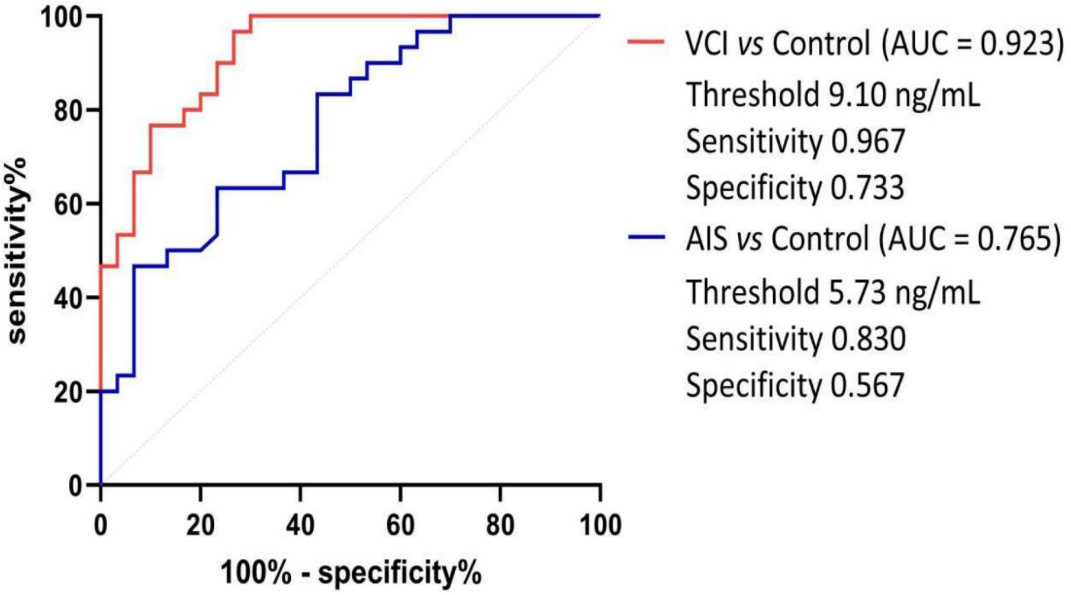

Supplementary Figure S6: ROC curves of GDF-15. ROC: receiver operating characteristic; GDF-15: growth differentiation factor 15; AIS: acute ischemic stroke; VCI: vascular cognitive impairment; AUC: area under the curve.
